# Supplementary material for: Sequential Ultrasound Assessment of Peri-Articular Soft Tissue in Adhesive Capsulitis of the Shoulder: Correlations with Clinical Impairments—Sequential Ultrasound in Adhesive Capsulitis
Source: Diagnostics (Basel). 2022 Sep 15;12(9):2231. doi: 10.3390/diagnostics12092231 (PMC9497809; doi:10.3390/diagnostics12092231)
Supplement: Supplementary file 1 [file diagnostics-12-02231-s001.zip › diagnostics-1876002-supplementary.pdf]

Table S1. STROBE Statement.

	Item No.	Recommendation	Page No.	Relevant text from manuscript
**Title and abstract**	1	(*a*) Indicate the study's design with a commonly used term in the title or the abstract	1	A prospective observational study of 56 patients
		(*b*) Provide in the abstract an informative and balanced summary of what was done and what was found	1	Abstract, line 32-34
**Introduction**				
Background/rationale	2	Explain the scientific background and rationale for the investigation being reported	P2	Introduction, line 46-52
Objectives	3	State specific objectives, including any prespecified hypotheses	P2	Introduction, line 60-65
**Methods**				
Study design	4	Present key elements of study design early in the paper	P2	Study Design and Participants, line 68
Setting	5	Describe the setting, locations, and relevant dates, including periods of recruitment, exposure, follow-up, and data collection	P2	Study Design and Participants, line 68-69<br>Baseline Demographics and Clinical Assessments, line 81-90
Participants	6	(*a*) *Cohort study*—Give the eligibility criteria, and the sources and methods of selection of participants. Describe methods of follow-up<br>*Case-control study*—Give the eligibility criteria, and the sources and methods of case ascertainment and control selection. Give the rationale for the choice of cases and controls<br>*Cross-sectional study*—Give the eligibility criteria, and the sources and methods of selection of participants	P2	Study Design and Participants, line 70-74
		(*b*) *Cohort study*—For matched studies, give matching criteria and number of exposed and unexposed<br>*Case-control study*—For matched studies, give matching criteria and the number of controls per case	N/A	N/A
Variables	7	Clearly define all outcomes, exposures, predictors, potential confounders, and effect modifiers. Give diagnostic criteria, if applicable	P3-4	Material and Methods, line 103-140

Data sources/ measurement	8*	For each variable of interest, give sources of data and details of methods of assessment (measurement). Describe comparability of assessment methods if there is more than one group	P3-4	Material and Methods, line 103-140
Bias	9	Describe any efforts to address potential sources of bias	P3	Ultrasound Parameter, line 106-107
Study size	10	Explain how the study size was arrived at	N/A	N/A

Continued on next page

Quantitative variables	11	Explain how quantitative variables were handled in the analyses. If applicable, describe which groupings were chosen and why	P4	Statistical Analysis, line 142-158
Statistical methods	12	(*a*) Describe all statistical methods, including those used to control for confounding	P4	Statistical Analysis, line 142-158
		(*b*) Describe any methods used to examine subgroups and interactions	P4	Statistical Analysis, line 142-158
		(*c*) Explain how missing data were addressed	P4	Statistical Analysis, line 148-150
		(*d*) *Cohort study*—If applicable, explain how loss to follow-up was addressed<br>*Case-control study*—If applicable, explain how matching of cases and controls was addressed<br>*Cross-sectional study*—If applicable, describe analytical methods taking account of sampling strategy	NA	NA
		(*e*) Describe any sensitivity analyses	NA	NA
**Results**				
Participants	13*	(a) Report numbers of individuals at each stage of study—eg numbers potentially eligible, examined for eligibility, confirmed eligible, included in the study, completing follow-up, and analysed	P5	Patient Characteristics, line 161-162
		(b) Give reasons for non-participation at each stage	P5	Patient Characteristics, line 161-162
		(c) Consider use of a flow diagram	P3	Figure 1
Descriptive data	14*	(a) Give characteristics of study participants (eg demographic, clinical, social) and information on exposures and potential confounders	P5	Patient Characteristics, line 161-169
		(b) Indicate number of participants with missing data for each variable of interest	P5	Patient Characteristics, line 161-162
		(c) *Cohort study*—Summarise follow-up time (eg, average and total amount)	N/A	N/A
Outcome data	15*	*Cohort study*—Report numbers of outcome events or summary measures over time		
		Case-control study—Report numbers in each exposure category, or summary measures of exposure	P5-6	Initial Values and Sequential Changes of Ultrasound Parameters, line 176-189
		Cross-sectional study—Report numbers of outcome events or summary measures		
Main results	16	(*a*) Give unadjusted estimates and, if applicable, confounder-adjusted estimates and their precision (eg, 95% confidence interval). Make clear which confounders were adjusted for and why they were included	P7	Correlations between UItrasound Parameters and Clinical Variables, line 222-227
		(*b*) Report category boundaries when continuous variables were categorized	N/A	N/A

	(*c*) If relevant, consider translating estimates of relative risk into absolute risk for a meaningful time period	N/A	N/A

Continued on next page

Other analyses	17	Report other analyses done—eg analyses of subgroups and interactions, and sensitivity analyses	N/A	N/A
**Discussion**				
Key results	18	Summarise key results with reference to study objectives	P8	Discussion, line 233-240
Limitations	19	Discuss limitations of the study, taking into account sources of potential bias or imprecision. Discuss both direction and magnitude of any potential bias	P9-10	Discussion, line 304-318
Interpretation	20	Give a cautious overall interpretation of results considering objectives, limitations, multiplicity of analyses, results from similar studies, and other relevant evidence	P10	Conclusion, line 320-327
Generalisability	21	Discuss the generalisability (external validity) of the study results	P9	Discussion, line 315-319
**Other information**				
Funding	22	Give the source of funding and the role of the funders for the present study and, if applicable, for the original study on which the present article is based	P10	Funding, line 333-336

*Give information separately for cases and controls in case-control studies and, if applicable, for exposed and unexposed groups in cohort and cross-sectional studies.

Note: An Explanation and Elaboration article discusses each checklist item and gives methodological background and published examples of transparent reporting. The STROBE checklist is best used in conjunction with this article (freely available on the Web sites of PLoS Medicine at http://www.plosmedicine.org/, Annals of Internal Medicine at http://www.annals.org/, and Epidemiology at http://www.epidem.com/). Information on the STROBE Initiative is available at www.strobe-statement.org.
